# Gait control in a soft robot by sensing interactions with the environment using self-deformation

**DOI:** 10.1098/rsos.160766

**Published:** 2016-12-07

**Authors:** Takuya Umedachi, Takeshi Kano, Akio Ishiguro, Barry A. Trimmer

**Affiliations:** 1 Graduate School of Information Science and Technology, The University of Tokyo, Takeda Bldg. Rm. 309, 2-11-16 Yayoi, Bunkyo-ku, Tokyo 113-0032, Japan; 2 Research Institute of Electrical Communication, Tohoku University, 2-1-1 Katahira, Aoba-ku, Sendai 980-8577, Japan; 3 Department of Biology, Tufts University, 200 Boston Avenue, Medford, MA 02155, USA; 4 Japan Science and Technology Agency, CREST, 7 Goban-cho, Chiyoda-ku, Tokyo 102-0075, Japan

**Keywords:** biologically inspired robot, decentralized control, soft-bodied robot, mechanosensing, behavioural diversity

## Abstract

All animals use mechanosensors to help them move in complex and changing environments. With few exceptions, these sensors are embedded in soft tissues that deform in normal use such that sensory feedback results from the interaction of an animal with its environment. Useful information about the environment is expected to be embedded in the mechanical responses of the tissues during movements. To explore how such sensory information can be used to control movements, we have developed a soft-bodied crawling robot inspired by a highly tractable animal model, the tobacco hornworm *Manduca sexta*. This robot uses deformations of its body to detect changes in friction force on a substrate. This information is used to provide local sensory feedback for coupled oscillators that control the robot's locomotion. The validity of the control strategy is demonstrated with both simulation and a highly deformable three-dimensionally printed soft robot. The results show that very simple oscillators are able to generate propagating waves and crawling/inching locomotion through the interplay of deformation in different body parts in a fully decentralized manner. Additionally, we confirmed numerically and experimentally that the gait pattern can switch depending on the surface contact points. These results are expected to help in the design of adaptable, robust locomotion control systems for soft robots and also suggest testable hypotheses about how soft animals use sensory feedback.

## 1. Introduction

Most animals are soft-bodied or proceed through life stages lacking a stiff skeleton. Even animals with stiff skeletons, such as adult insects and mammals, are mainly composed of liquids and soft tissues such as muscles, tendons and layers of skin-like tissues [1]. These soft materials are critical for many characteristics of animal locomotion including energy efficiency, flexibility, adaptability, multi-functionality, self-healing and stability [2–6]. Animals without stiff skeletons are also able to change size and shape and access restricted environments. Such animals interact extensively with the environment (e.g. storing and releasing elastic energy) and can even use the mechanical properties of a substrate to modify their locomotion strategy (e.g. the ‘environmental skeleton’ [7,8]). Because soft terrestrial animals are typically in continuous contact with their environment, mechanosensory feedback is expected to be important for making their locomotion effective and adaptive.

In contrast with their widespread distribution in animals, soft materials are not used extensively in the mechanical design of machines; even robots inspired by caterpillars [9,10] and earthworms [11,12] are generally designed with hard and rigid materials. Although incorporating soft materials into robots could provide animal-like capabilities [13–16], soft materials deform into complex shapes (twisting, buckling, wrinkling and so on) easily in three-dimensional space which makes them difficult to control using conventional robotics approaches. Even during the design process, modelling and predicting such soft-bodied motion require vast amounts of computation because of the unlimited degrees of freedom, nonlinear responses of the materials, intermittent changes in the boundary condition (e.g. friction and contact faces), and large deformations that cannot be described properly by solid mechanics. Hence, it is extremely difficult to understand such motion within the framework of the traditional centralized control scheme.

**Figure 1.**
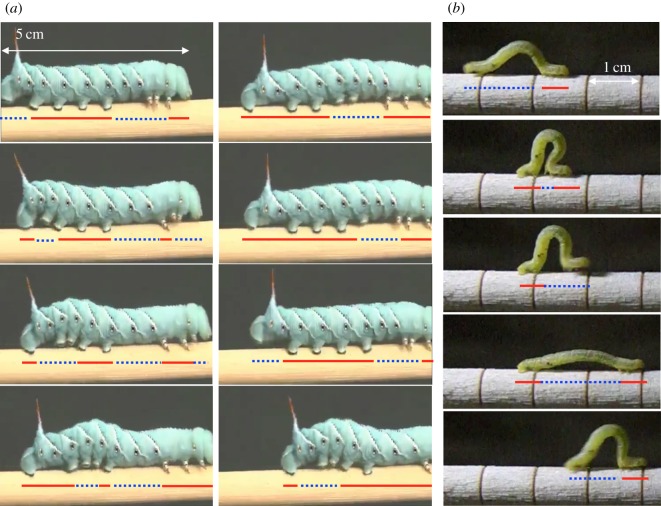
(*a*) Crawling and (*b*) inching locomotion gaits of soft-bodied animals (caterpillars) that lack a hard skeleton and generate adaptive behaviours in complex environments without massively complex brains. Note the arrangement of prolegs on the mid-body segments in (*a*) *Manduca sexta* that are lacking in the inching caterpillar (*b*). The red solid lines indicate gripping segments whereas the blue dashed lines indicate non-gripping segments.

One approach to addressing this problem is to look at the control mechanisms used by relatively simple and biologically well-known soft animals, whose processes have evolved over millions of years to produce effective locomotion without massively complex brains [17,18]. Using this perspective, we have studied a highly tractable animal model, the tobacco hornworm *Manduca sexta*, and other caterpillar species (figure 1) to understand how animals that lack a hard skeleton can coordinate their motion. Caterpillars generate resilient and adaptive behaviour in three-dimensional space; not only can these bodies stretch or compress they can also bend, wrinkle, buckle, twist, droop and creep along or against complex environments [19]. Furthermore, caterpillars differ in their morphological characteristics such as their weight, arrangements of prolegs,^1^ and shape, and they use a variety of distinct gaits [20]. Considering their small numbers of neurons and the complexity of their body dynamics, it is conceivable that motion control is achieved through autonomous decentralization in which mechanical interactions and locally distributed signalling organize cohesive movements.

In this context, soft structures could play an important sensory role to monitor complex motion and also to compensate for limited central computational capacities [21]. An interesting, and largely unexplored, aspect of highly deformable animals is how they collect sensory information for use in motor control. In general, mechanosensing for locomotion (as distinct from acoustic sensing) in animals consists of two systems. One involves a variety of strain sensors on the surface of the body that are specialized for collecting information about the environment and are collectively referred to as the sense of touch. The other system senses changes in the strain, relative position, or forces exerted within the body and this is referred to as proprioception [22]. Information from these two sensory systems is usually processed in different regions of the central nervous system. However, in soft animals the distinction between these modalities is not well understood as external forces will easily deform the body and are expected to have a significant impact on both tactile and proprioceptive receptors. For example, when filiform tactile sensors on the body surface of *Manduca sexta* are in contact with the environment they are inevitably activated by movements of the caterpillar itself [23]. Similarly, the response properties of some proprioceptive receptors such as the stretch-receptor organs in *Manduca sexta* suggest they are not well suited for real-time sensing of segment length [24] but they are activated by external forces [25]. It is therefore possible that by sensing self-deformation an animal such as *Manduca sexta* is able to collect critical information about its interaction with the environment. This could include characteristics such as friction that are very difficult to predict or model by conventional methods.

The goal of this research is to understand how interactions between a moving soft body and its environment can provide useful sensory information for controlling locomotion in a highly deformable robot. For this purpose we have focused on how body deformations resulting from friction forces can serve as a local sensory signal to control movement produced by a coupled oscillator system. Each oscillator drives rhythmic contraction of a segment. The oscillators modify their phase in response to leg deformations as the robot interacts with its environment. Hence, robot motion serves to couple the oscillators (decentralized controllers) to mechanosensory feedback. We show that, although the material and structural properties of the body are complex, by monitoring a single sensory value of the resulting deformation it is possible to generate appropriate phase differences for locomotion. Furthermore, we confirmed numerically and experimentally that the gait pattern can switch between inching and crawling appropriately when the surface contact points are changed. These gaits and differing morphologies have correlates in a variety of caterpillar species suggesting that such findings could help to understand the locomotion control strategies of some soft animals.

The remainder of this paper is organized as follows. Section 2 first introduces a mathematical model that represents the body designs of real crawling and inching caterpillars. The model consists of a series of oscillators with coupled mechanical outputs that together form a decentralized control system. This is implemented in a numerical simulation that produces organized locomotion. Based on the findings from the numerical experiments, §3 then presents a hardware implementation of the system in a modular robot. This robot is used to collect experimental data showing how two distinct caterpillar-like gaits (inching and crawling) can be produced simply by altering the local friction feedback conditions. Section 4 discusses the importance of the mechanosensory feedback from the substrate with numerical experiment, the differences between the numerical and experimental results, and the implications of these results for the evolution of different caterpillar gaits. Finally, the paper concludes in §5 with a summary of the present work and proposed future work.

## 2. Mathematical model

This section is for extracting the control mechanism from two distinct caterpillars (crawling and inching ones). To this end, we first model the deformable structure and motion control mechanism of the real caterpillars. The goal here is to keep the mathematical model as simple as possible but still mimic the relationship between the segment length and gripping/releasing mechanism of the leg.

### 2.1. Dynamics of the mechanical system

**Figure 2.**
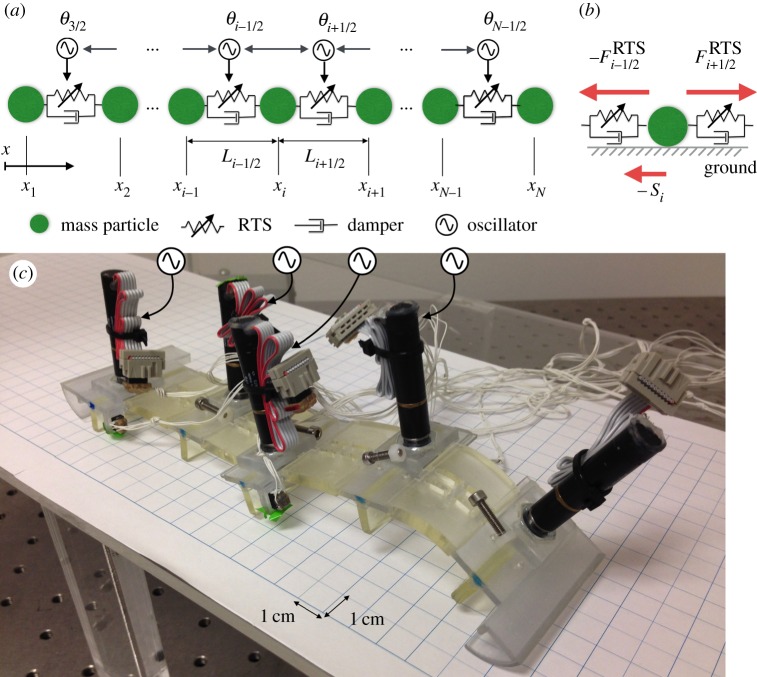
(*a*) Schematic of a mechanical model of the caterpillar-like soft robot. Double-headed arrows between the oscillators indicate diffusion interaction. Arrows from the oscillators to real-time tunable springs (RTSs) represent the motor command to RTSs. (*b*) Forces acting on a mass of the model. (*c*) The three-dimensionally printed caterpillar-like soft-bodied robot consisting of four segments.

We model the mechanical system of a caterpillar as a linear chain of masses linked by real-time tunable springs (RTSs),^2^ as can be seen in figure 2*a*. All parameters and variables of the model are listed in table 1. RTS is a muscle-inspired passive actuator that can change its resting length dynamically with stiffness *k* and linear damping coefficient *c* (both of them are fixed values). This can be implemented in hardware as explained later. Position of the mass, *x_i_* (*i *= 1−*N*), is governed by the following equation:

2.1 $$m{\ddot{x}}_{i}=-F_{i-1/2}^{\mathrm{RTS}}+F_{i+1/2}^{\mathrm{RTS}}-s_{i},$$

where $-F_{i-1/2}^{\mathrm{RTS}}$ and $F_{i+1/2}^{\mathrm{RTS}}$ are the force from the RTSs linked with mass *i*, and −*s_i_* is the friction force from the ground on mass *i* (figure 2*b*).

**Table 1.** Parameters and variables of the robot.

variables	explanation	
physical variables of segment *i*		
*x_i_*	position of mass *i*	equation (2.1)
$L_{i+1/2}$	resting length of the RTS between mass *i* and mass *i + *1	equation (2.2)
$F_{i}^{A}$	force from a RTS between mass *i* and mass *i + *1	equation (2.3)
*s_i_*	ground reaction force stemming from shear stress on the segment	equation (2.5)
*α_i_*	coefficient that defines gripping capability on mass *i*	figure 3
parameters and variables of the control system (oscillators)		
$\theta_{i+1/2}$	phase of oscillator i+1/2	equation (2.6)
$I_{i+1/2}$	discrepancy function on RTS i+1/2	equation (2.7)
parameters		
*m*	mass of mass *i*	0.3 g
*k*	stiffness the RTSs	100.0 g cm s^−2^
*c*	linear damping coefficient of the RTSs	1.0
$\bar{L}$	maximum length of the resting length variation of RTSs	0.5 cm
*A*	coefficient that specifies amplitude of the resting length variation of RTSs	0.3
*α_i_*	coefficient that defines gripping capability on mass *i*	figure 3
*ω*	intrinsic frequency of oscillator i+1/2	3.14
*ε*	diffusion coefficient between neighbouring oscillators	0.2
*σ*	strength of the local sensory feedback	0.2

### 2.2. Segment contraction/extension driven by the real-time tunable spring

The model is driven by resting length variations of the RTSs, which are controlled with the phase of the oscillator, $\theta_{i+1/2}\;(0\leq \theta_{i+1/2}<2\pi )$ (the dynamics is explained in the following subsection). The waveform of the resting length variation can be designed arbitrarily as any function. In this mathematical model, we set the waveform as follows:

2.2 $$L_{i+1/2}=\bar{L}(1+A(\cos\theta_{i+1/2}-1)),$$

where $\bar{L}$ is the maximum resting length and *A* specifies the amplitude. Hence, the force from RTS between mass *i* and mass *i *+ 1 on mass *i* is written by

2.3 $$F_{i+1/2}^{\mathrm{RTS}}=k(l_{i+1/2}-L_{i+1/2})+c{\dot{l}}_{i+1/2},$$

where $l_{i+1/2}$ is the actual length of RTS between mass *i* and mass *i *+ 1 (i.e. $l_{i+1/2}=x_{i+1}-x_{i}$).

### 2.3. Gripping mechanism

To simplify three-dimensional caterpillar locomotion to one-dimensional motion of a linear chain of masses linked by RTSs, we model the gripping mechanism as the following friction force:

2.4 $$s_{i}=\mu_{i}{\dot{x}}_{i},$$

where *µ_i_* is the friction coefficient described with viscous friction. As can be seen in figure 1, legs on the real caterpillar grip when the body segment elongates and the legs are in contact with the substrate. Meanwhile, legs can release the substrate as the segment contracts. To model the gripping mechanism with a one-dimensional model, we designed the friction to change depending on the actual length of the RTS(s) (which is represented by ${\tilde{l}}_{i}$) and by the presence of legs^3^ (which is defined by *α_i_*) that can contact the ground. This is given by

2.5 $$\mu_{i}=\alpha_{i}{\tilde{l}}_{i},$$

where ${\tilde{l}}_{i}=1/2(l_{i-1/2}+l_{i+1/2})$ (2 < *i* < *N* − 1), ${\tilde{l}}_{1}=l_{3/2},{\tilde{l}}_{N}=l_{N-1/2}$, and *α_i_* is a coefficient (more than or equal to 0), which determines the presence of a leg. When *α_i_* > 0, the body segment has legs that grip the ground when the segment is extended (i.e. ${\tilde{l}}_{i}$ becomes long) and the equation roughly models this with an increasing friction coefficient, *µ_i_*. On the other hand, the friction coefficient, *µ_i_*, decreases when the segment contracts (i.e. ${\tilde{l}}_{i}$ becomes short). This corresponds to the lifted-up segment and legs release of the real caterpillar. In the case of *α_i_* = 0, we assume that the body segment does not have legs (no touch nor friction with the ground).

### 2.4. Control system

Caterpillar locomotion can be classified into two distinct locomotion forms: crawling and inching [19] (figure 1). Larger caterpillars tend to have prolegs in the middle segments and to use crawling locomotion. In the locomotion form, a wave of steps is initiated at the posterior, and the wave is transmitted to front segments. This bending and contrasting wave pattern from the rear to front is called ‘anterograde wave’. Smaller caterpillars tend to lack prolegs in the middle segments and to produce inching locomotion. In inching locomotion, the posterior legs are pulled forward to grip the substrate just behind the front (thoracic) segments, the front grip is released, and then the body is extended forward. This locomotion form also requires an anterograde wave in the sense that the first step is started from the rear and then sent to the front.

The unique point of this proposed model is local sensory feedback attained from the interaction between motion of the segments and substrate, which allows the oscillators to produce the anterograde oscillatory pattern for locomotion. To control the resting length of the RTS, we designed the dynamics of the oscillators based on a ‘discrepancy function’ as

2.6 $${\dot{\theta }}_{i+1/2}=\omega +\varepsilon \underset{j=i-1/2,i+3/2}{\sum }\sin(\theta_{j}-\theta_{i+1/2})-\frac{\partial I_{i+1/2}}{\partial \theta_{i+1/2}},$$

where *ω* is the intrinsic frequency of the oscillator, the second term on the right-hand side describes diffusion interactions between neighbouring oscillators (with diffusion coefficient *ε*), and the third term is the local sensory feedback based on the discrepancy function, a measure of *the undesirable difference* recorded by the local sensor and the controlled (target) values. Without the third term, it is obvious that the all phase converse to in-phase condition due to the diffusion interactions of the second term. So the third term is a key to generate an anterograde phase pattern.

To design a local sensory feedback system, we introduce a new design scheme to use friction force locally sensed on a body segment produced by touch between the body segment and environment during the locomotion. Through our observations of locomotion, we hypothesize that many animals *evaluate friction forces from the environment in real time and change the reaction against it*, i.e. increasing or decreasing the friction by changing surface condition (e.g. with leg, mucus, and surface deformation) for producing propulsion in a desired direction. In the particular case of caterpillar locomotion, the body segment should hold the substrate when it feels backward friction, whereas the body segment should release the ground when it feels forward friction. Hence, we focus on friction force (sensor value) *s_i_*, and $L_{i+1/2}(\theta_{i+1/2})$ (resting length of RTS, i.e. body segment) to regulate propulsion in a desired direction for a decentralized controller. A discrepancy function can be designed as

2.7 $$I_{i}=\sigma \cdot s_{i}\cdot L_{i+1/2}(\theta_{i+1/2}),$$

where *σ* specifies the strength of the local sensory feedback. An important feature is that an extended body segment *i* is needed to increase the friction coefficient, *µ_i_* (equation (2.5)). When *s_i_* is positive (mass *i* moving forward), high friction coefficient (large length of the segment) is undesirable because the segment drags and prevents the other segments' forward locomotion. In this case, the longer the resting lengths $L_{i+1/2}(\theta_{i+1/2})$ the higher the value of *I_i_*. Meanwhile, when *s_i_* is negative (mass *i* moving backward), small friction coefficient (shorter length of the segment) is undesirable because the segment cannot hold the ground and support the other segments moving forward. In this case, the shorter the resting lengths $L_{i+1/2}(\theta_{i+1/2})$ the higher the value of *I_i_*. The third term in equation (2.7) works to tune the angular frequency dynamically so as to avoid higher value of *I_i_*. Consequently, the phase is mainly modulated to pull towards 0 when *s_i_* < 0, which in turn increases the resting length $L_{i+1/2}$ (equation (2.2)) and the friction coefficient *µ_i_* (equation (2.5)). On the other hand, the phase is mainly modulated to pull towards *π* when *s_i_* > 0, which in turn decreases the resting length $L_{i+1/2}$ and friction coefficient *µ_i_*.

### 2.5. Numerical experiment

**Figure 3.**
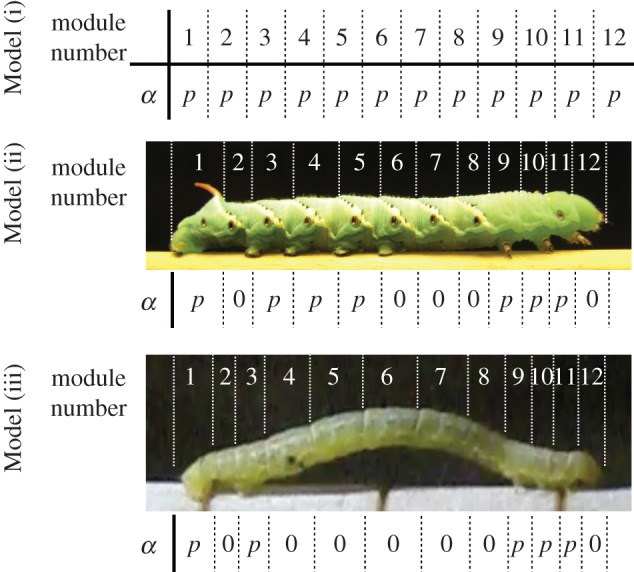
Parameter set-ups of the coefficients that define the gripping capability of mass *i* in Models (i), (ii) and (iii). The top set-up (Model (i)) is tested for verifying the mathematical model. The last two set-ups (Model (ii) and Model (iii)) correspond to crawling and inching caterpillars, respectively.

**Figure 4.**
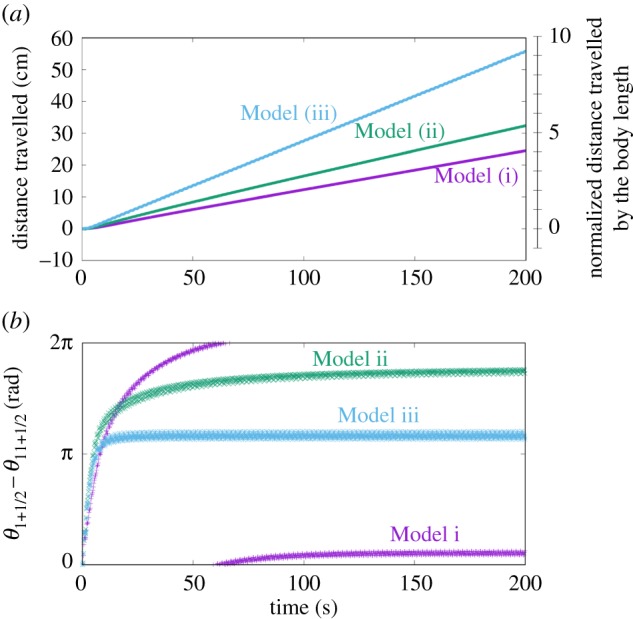
(*a*) The cumulative distance travelled of Models (i), (ii) and (iii) and (*b*) phase difference between the front and rear oscillators.

**Figure 5.**
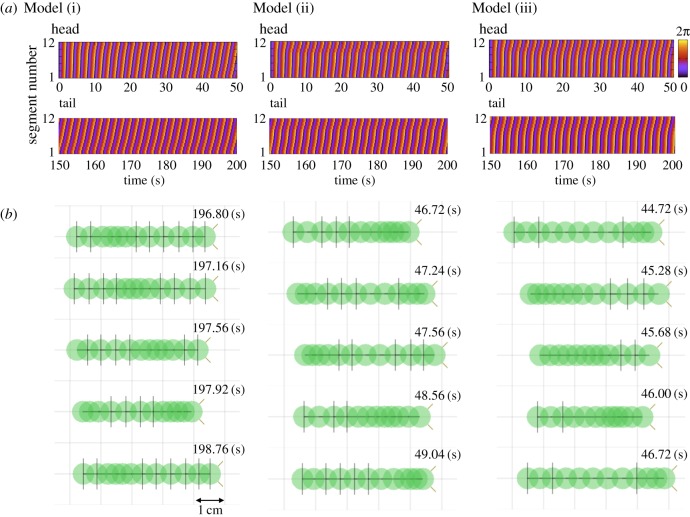
Time evolution of phase of the oscillators (*a*) and snapshots of one cycle of the locomotion (*b*) in Model (i), Model (ii) and Model (iii). The green circles present positions of the masses. The lines passing through the centre of the circle vertically indicate high friction segments. The segment with the tentacles (two light brown slanting lines) indicates the head.

To validate this control scheme, we conducted a numerical simulation. The number of modules, *N*, is set as 12, which corresponds to the body structures of the real crawling and inching caterpillars (figure 3). *α_i_* is set as a positive coefficient, *p*, when the segment has prolegs, whereas *α_i_* is set as zero when the segment does not have prolegs. It is known that gripping system of the real caterpillars is very sophisticated: only one pair of prolegs is good enough to support the entire body of the real caterpillar [20]. Hence, we assume value of *p* should be sufficiently high for the model and set *p* to 10 in this section. We will discuss how decreasing the value effects the model in the Discussion section. Three different sets of leg arrangement are prepared as Models (i), (ii), and (iii) (figure 3). All models produce consistent locomotion, measured by the cumulative distance travelled (figure 4*a*) and the near stability of the oscillator phase (figure 4*b*). The details of these gaits are shown in figure 5.

Model (i) is a mathematical model in which all segments have legs. This produced the most marked posterior to anterior phase shift (approx. 2*π*; figure 5) and the slowest locomotion. Given that the second term (diffusion term) in equation (2.6) serves to produce in-phase oscillation with the neighbouring oscillators, the local sensory feedback (the third term in equation (2.6)) provides the phase shift for generating crawling locomotion. What is happening locally is that the local sensory feedback modifies the own phase so as to increase the ground friction when it moves backward whereas so as to decrease the ground friction when it moves forward (as equations (2.5)–(2.7) indicate).

Model (ii) corresponds to caterpillars such as *Manduca sexta* which are thought to represent the ancestral configuration with prolegs in the middle of the body. For these animals, locomotion usually involves a compression wave proceeding from the rear to the head accompanied by a lifting motion away from the substrate (crawling, figure 1*a*). Model (ii) produced a similar crawling gait characterized by an anterograde phase shift (the posterior to anterior phase shift is approx. 2*π*) of the contraction–relaxation cycles from the rear to head and an intermediate speed.

Model (iii) corresponds to the anatomy of relatively smaller caterpillars such as geometrids which have no proleg in the middle of the body and tend to contract and lift up the middle segments together (inching, figure 1*b*). Model (iii) produced a similar inching gait with contraction–relaxation cycles in the middle segments nearly in-phase (the posterior to anterior phase shift is approx. *π*) and the fastest locomotion.

Overall, phase shift from the rear to front of the anterograde wave depends on the proleg arrangements. We will discuss further these results and compare them with real animals in §4. It should be noted that the oscillators of the segments ‘without prolegs’ (i.e. without the local sensory feedback) in Models (i) and (ii) oscillate nearly in-phase between the neighbouring oscillators (figure 5). The results also indicate that the same number of segments is not necessary when we design the prototype.

## 3. A physical soft-bodied robot

Based on the findings from the numerical experiments, we build a prototype of the model. The purpose is not for imitation of the real caterpillar but for validation of the extracted control mechanism from the mathematical model. Needless to say, the state-of-art robotics technology has still many limitations compared with the living system (e.g. power-to-weight ratio compared with muscle, microfabrication technology, energy efficiency of actuators, and energy storage capacity of batteries). Therefore, we downsize the 12-segmented mathematical model to 4-segmented prototype.

### 3.1. The hardware design

**Figure 6.**
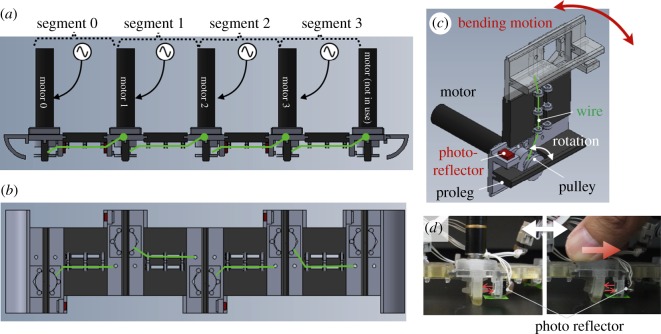
(*a*) Side view and (*b*) bottom view of CAD image of the entire robot composed of four segments. (*c*) CAD image of one segment. The black parts are three-dimensionally printed with rubber-like materials (except for the motors) whereas the grey parts are three-dimensionally printed with hard and rigid materials. The green lines indicate wires that are wound and unwound by the motors in the segment. (*d*) Photos describe how to detect the deformation of the proleg with the photoreflector.

The caterpillar-like soft robot consists of four segments shown in side and bottom views (figures 2*c* and 6*a*,*b*). Figure 6*c* shows a bird's eye view of a CAD image of one segment, which consists of a DC motor with encoder (RE10: 256102, with gearhead GP10A: 218416, and encoder MRenc Type S: 201933; Maxon Motor ag, Sachseln, Switzerland), a pulley, and the deformable beam structure of the robot segment. The wire (nylon fishline) is wound and unwound by the pulley, which generates bending motion of the beam. This motor-tendon actuator produces active tensile force and is therefore analogous to a muscle. The black parts (except for the motors) were three-dimensionally printed from a rubber-like polymer (Objet Fullcure® 930 TangoPlus), whereas the grey parts were printed with hard material (VeroClear). These parts are directly printed at once using a multimaterial printer (Objet, Connex 500). For convenience, the five ground contact points are called ‘prolegs’ because of their correspondence to the caterpillar gripping system [26,27].

The prolegs have two roles: they exert friction when the body segment elongates in contact with the ground; and they indirectly report the fiction force between the proleg and ground. Arc structures were attached to the leading and trailing edges of the robot to allow the prolegs to lift when the segments contracted (figure 6*a*). The arcs were printed with hard smooth material which was slippery and able to slide over the ground with little force. When the proleg (printed with rubber-like material, figure 6*c,d*) is in contact with the ground, horizontal movement of the segment causes shear stress between the motor mount and the ground (see right photo in figure 6*d*). The friction force resisting segment movement causes proleg deformation that is sensed with a photoreflector (QRD1114, Fairchild Semiconductor International, Inc., San Jose, CA, USA; figure 6*c*,*d*). The deformation is released when the segment contracts and is lifted up. This deformation roughly corresponds to the friction force of the segment as described in the mathematical model.

The RTS is produced by the back-drivable motor controlled using the phase oscillator (computed in the micro-controller, Mbed LPC1768, NXP Semiconductors, Eindhoven, The Netherlands) to wind and unwind the wire. The length of the remaining wire can be measured by the encoder and the resting length of the RTS is controlled using proportional--differential control. The target pulley angle (which determines the resting length) is changed according to the phase, $\theta_{i+1/2}$, of the oscillator (equation (2.6)).

In the robotic model, we change the resting length of RTS variation as follows:

3.1 $$L_{i+1/2}=\left\{ \begin{array}{l} \bar{L}(1+A(\cos2\theta_{i+1/2}-1))\;\mathrm{when}\;0\leq \theta_{i+1/2}<\pi ,\\ \bar{L}\;\mathrm{otherwise}. \end{array}\right.\;$$

The contraction was stopped while $\pi \leq \theta_{i+1/2}<2\pi$ to remove residual strain accumulated in the deformable beam of the segment. Using this equation, the local sensory feedback can be calculated according to equations (2.6) and (2.7), which does not differ essentially from the mathematical model.

### 3.2. Experimental results with the robot

To test the practicality of the mathematical model, we replicated two of the simulation configurations (crawling and inching) on the robot. The crawling configurations correspond to Model (i) or Model (ii), in which each segment can receive local sensory information from deformation of the corresponding body part. By contrast, the inching configuration corresponds to Model (iii), in which segments 1 and 2 do not provide sensory information. To replicate these arrangements, smooth (low friction) tape was attached to the prolegs in segments 1 and 2 and the resting length of RTS in segment 1 was set shorter than those of the other segments to lift the prolegs of segments 1 and 2. This set-up is equivalent to setting values of *α*_1_ and *α*_2_ as 0 in the mathematical model. The robot is placed on a copy paper which is glued on a levelled table.

**Figure 7.**
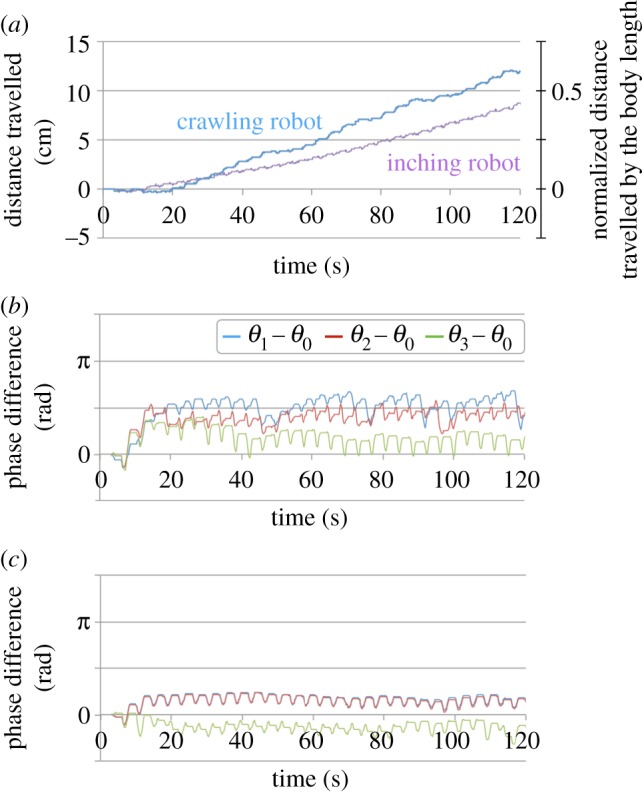
(*a*) Cumulative distance travelled of the crawling robot and inching robot. Time evolution of phase difference in the crawling robot (*b*) and inching robot (*c*). Comparing data with the simulations is shown as *θ*_1_ − *θ*_0_.

Locomotion was produced in each of these configurations (figure 7*a*) and the oscillators successfully generate anterograde oscillatory pattern (figure 7*b*,*c*). It is notable that phase shift between the oscillators is amended so as to maintain anterograde oscillatory pattern every time the phase shift is perturbed: phase relation between *θ*_1_ − *θ*_0_ and *θ*_2_ − *θ*_0_ becomes inverse around 80 and 95 s in figure 7*b* but is recovered to an anterograde oscillatory pattern afterward.

This self-recovering of the phase relation is achieved by the local sensory feedback with the soft body. Considering that the robot form is a continuum and deformable beam with friction on ground, once upward bending occurs, it can be easily sent to the peripheral segments (like a wave motion of a rope on a ground). The proposed feedback controls the direction of the wave motion, which also produces an anterograde wave eventually.

**Figure 8.**
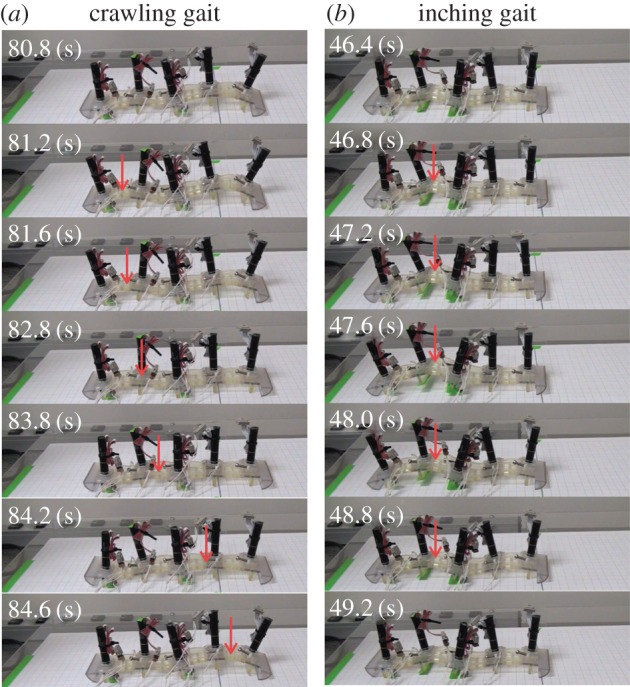
Snapshots of one locomotion cycle for the crawling robot (*a*) and the inching robot (*b*). The red arrows indicate the peak of bending deformation activated with RTS contraction.

Tracking movements showed that crawling and inching locomotion were distinguished by the deformation peak which was transferred from the rear to the front in the crawling configuration but remained in the middle of the body in the inching configuration (figure 8). This difference was visible in the phase gradient from the rear to the front that arose in the crawling robot (figure 7*b*) whereas in-phase contraction of segments 1 and 2 is seen in inching locomotion.

Interestingly, the locomotion performance differed from the numerical results: the crawling robot was faster than the inching robot (see Discussion) and the rear oscillator was the most phase delayed. This was probably caused by proleg deformation on the edge segment being bigger than those of the other segments.

## 4. Discussion

These results show that local sensory feedback encoding shear sensing of the robot's interaction with its environment can automatically generate locomotion. Which ‘gait’ is produced is a function of the overall ‘anatomy’ of the robot: with legs on each segment the robot crawls but when grip is eliminated in the mid-body segments, it inches. This corresponds to the typical anatomical configurations seen in species of crawling caterpillars such as *Manduca sexta,* and those that inch, such as the geometrids [19]. It has been hypothesized that both gaits could be generated by similar patterns of neural activity with differences in grip location accounting for the different movements [20].

**Figure 9.**
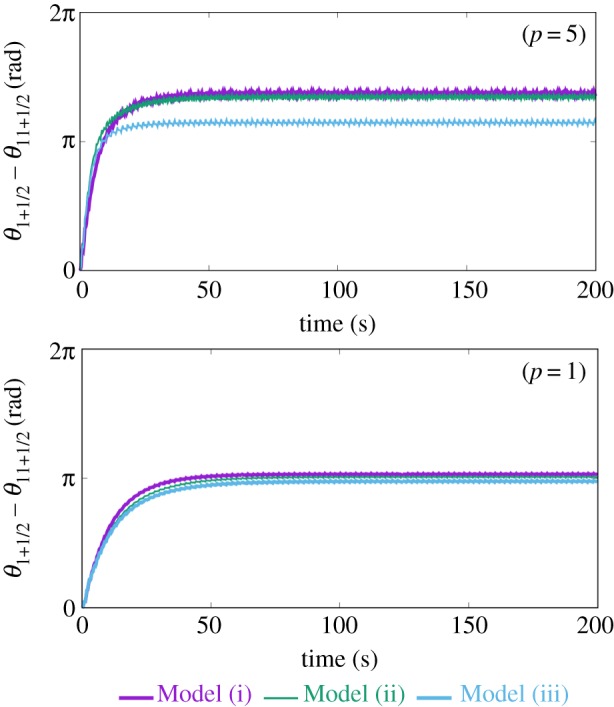
Phase difference between the front and rear oscillators in Models (i), (ii) and (iii) when changing *p* (that defines a friction coefficient of the legged segments).

The results reported here, from both simulations and physical implementation of a local sensing control system, suggest that internal motor programmes (central pattern generators [28,29]) could automatically reconfigure to produce crawling and inching. Such switching would require local information about the interaction of the animal with its substrate. To discuss further about the importance of the local mechanosensory information, we simulated less interactive circumstance between the model and its substrate by decreasing the value of *p*.^4^ Figure 9 represents phase gap from the rear to front oscillators with smaller values of *p*. The plots indicate that crawling and inching switching (the distinct gap from the rear to head between Models (ii) and (iii)) disappears with less friction from the substrate. This simulation result indicates that a real caterpillar may not be able to produce appropriate locomotion gait in the circumstance where interaction between the body and environment is not enough. One of the most extreme cases is placing *Maduca sexta* on a soft substrate: the animal cannot generate gait pattern and stop locomotion on the soft substrate.

The local information is not necessarily to be shear sensing. It is possible that touch sensors such as filiform hairs [30] or other types of strain sensors such as multi-dendritic neurons in the body wall [31–34] provide appropriate information. This is directly testable using genetic and pharmacological manipulations of the sensory cell activity (e.g. [35]).

The simulation results have many features in common with caterpillars. For example, Model (iii), the inching mode, is significantly faster than the others (figure 4*a*). This is because in-phase contractions of the middle segments allow the structure to extend and contract over a larger distance, effectively producing a long ‘step length’. Providing that grip is adequate, long steps will always produce faster locomotion than small steps at a given cycle frequency. In general, the locomotion speed of inching caterpillars with secured gripping (normalized by the body length) is faster than that of crawling caterpillars [19].

There are still questions that remain, particularly in the differences between simulation results and the performance of the physical robots. For example, why is the inching robot slower than the crawling robot? Our observations suggest that this is an artefact of the robot design. Because of the weight of the motors the robot cannot lift the middle of the body in the same way that inching caterpillars can. Most inching caterpillars are small and thin with a low mass in the centre of the body [20]. In addition, caterpillars have an extraordinarily effective gripping system [36,37] that prevents slippage and toppling even when the body is lifted high off the ground; the current robot cannot match either of these requirements.

## 5. Conclusion

This study presents an autonomous decentralized control that switches spontaneously its locomotion gait between crawling and inching gaits based on the locally available sensory information collected by the superficial deformation of the soft-bodied robot. Inspired by caterpillars, we designed a coupled oscillator system as the decentralized controller with local sensory feedback on the basis of the deformation of the leg of the soft-bodied robot. Through the designing process, we introduce how to install the sense of touch into the decentralized controller so as to allow the model/robot to exploit the friction force from the ground for generating locomotion. We also confirmed that both the mathematical model and the physical robot switch their gait pattern (i.e. crawling and inching) appropriately when the morphology is changed (i.e. an existence of prolegs and sensory inputs from the mid-segments). The results correspond to the morphological difference between crawling and inching caterpillars, which indicates that the animals also use a similar motion control regardless of the morphological difference. These results can also shed more light on the basic principles of soft-bodied biological systems.

## Supplementary Material

Supplementary material from “Gait control in a soft robot by sensing interactions with the environment using self-deformation”

## Footnotes

1 A proleg is a fleshy leg that develops on an abdominal segment of some insect larvae, which disappear in the adult. (They are confirmed in the middle and rear segments in figure 1*a* and the rear segments in figure 1*b*.)

2 We have developed such passively deformable actuator called RTS. For instance, we developed a coil spring that can alter its resting length forcibly wound/unwound by a DC motor [17]. Passivity can be emulated by a DC motor with proportional–derivative control, which is used in this paper.

3 Ones of which on the ventral surface of the abdomen of caterpillars are called prolegs.

4 Value of *p* influences the friction coefficient, *µ* (accompanying the segment length, RTS length), as equation (2.5) indicates, and of course friction force from the ground (*s_i_*, equation (2.4)).

## Data accessibility

All data used in this research are included in figures, table and electronic supplementary material. The simulation movie, experimental movie using the prototype, and the simulation code are also available in the electronic supplementary material.
